# Semi-quantitative MRI biomarkers of knee osteoarthritis progression in the FNIH biomarkers consortium cohort − Methodologic aspects and definition of change

**DOI:** 10.1186/s12891-016-1310-6

**Published:** 2016-11-10

**Authors:** Frank W. Roemer, Ali Guermazi, Jamie E. Collins, Elena Losina, Michael C. Nevitt, John A. Lynch, Jeffrey N. Katz, C. Kent Kwoh, Virginia B. Kraus, David J. Hunter

**Affiliations:** 1 Quantitative Imaging Center, Department of Radiology, Boston University School of Medicine, FGH Building, 3rd floor, 820 Harrison Avenue, Boston, MA 02118 USA; 2 Department of Radiology, University of Erlangen-Nuremberg, Erlangen, Germany; 3 Orthopaedic and Arthritis Center for Outcomes Research, Department of Orthopedic Surgery, Brigham and Women’s Hospital, Boston, MA USA; 4 Harvard Medical School, Boston, MA USA; 5 Department of Epidemiology and Biostatistics, University of California at San Francisco, San Francisco, CA USA; 6 University of Arizona Arthritis Center & Division of Rheumatology, University of Arizona College of Medicine, Tucson, AZ USA; 7 Duke Molecular Physiology Institute and Division of Rheumatology, Department of Medicine, Duke University School of Medicine, Durham, NC USA; 8 Department of Rheumatology, Royal North Shore Hospital and Institute of Bone and Joint Research, Kolling Institute, University of Sydney, St Leonards, NSW Australia

**Keywords:** Osteoarthritis, MRI, Progression, Scoring, Biomarkers

## Abstract

**Background:**

To describe the scoring methodology and MRI assessments used to evaluate the cross-sectional features observed in cases and controls, to define change over time for different MRI features, and to report the extent of changes over a 24-month period in the Foundation for National Institutes of Health Osteoarthritis Biomarkers Consortium study nested within the larger Osteoarthritis Initiative (OAI) Study.

**Methods:**

We conducted a nested case–control study. Cases (*n* = 406) were knees having both radiographic and pain progression. Controls (*n* = 194) were knee osteoarthritis subjects who did not meet the case definition. Groups were matched for Kellgren-Lawrence grade and body mass index. MRIs were acquired using 3 T MRI systems and assessed using the semi-quantitative MOAKS system. MRIs were read at baseline and 24 months for cartilage damage, bone marrow lesions (BML), osteophytes, meniscal damage and extrusion, and Hoffa- and effusion-synovitis. We provide the definition and distribution of change in these biomarkers over time.

**Results:**

Seventy-three percent of the cases had subregions with BML worsening (vs. 66 % in controls) (*p* = 0.102). Little change in osteophytes was seen over 24 months. Twenty-eight percent of cases and 10 % of controls had worsening in meniscal scores in at least one subregion (*p* < 0.001). Seventy-three percent of cases and 53 % of controls had at least one area with worsening in cartilage surface area (*p* < 0.001). More cases experienced worsening in Hoffa- and effusion synovitis than controls (17 % vs. 6 % (*p* < 0.001); 41 % vs. 18 % (*p* < 0.001), respectively).

**Conclusions:**

A wide range of MRI-detected structural pathologies was present in the FNIH cohort. More severe changes, especially for BMLs, cartilage and meniscal damage, were detected primarily among the case group suggesting that early changes in multiple structural domains are associated with radiographic worsening and symptomatic progression.

## Background

Knee osteoarthritis (OA) is a major public health concern with current treatment focusing on controlling symptoms since there are no interventions that have yet been approved for modifying the course of the disease or improving structural alterations in joint tissues [1]. The Foundation for the National Institutes of Health (FNIH) sample was selected for a nested case–control study designed to evaluate the predictive validity of a broad spectrum of imaging and biochemical markers of disease progression in knee OA derived from the Osteoarthritis Initiative (OAI) public data base, an ongoing multi-center prospective observational cohort study of knee OA [2]. A biomarker that exhibits change over the near-term and is associated with longer-term clinically important outcomes would have potential as a marker of treatment efficacy [2].

While radiography depicts structural bony tissue changes only in advanced stages of OA, magnetic resonance imaging (MRI) is able to visualize all involved joint tissues, even in the earliest stages of disease, in which radiographs are normal [3, 4]. Recent data suggest that non-cartilaginous tissue changes in particular play an important role in the onset and progression of osteoarthritis [5, 6].

Using multivariable logistic regression models to examine associations between structural MRI markers and progression of radiographic and pain outcomes, we showed recently that all baseline structural joint features with the exception of effusion-synovitis and meniscal morphology, were able to predict 48 month case status and that for all joint features evaluated including size of bone marrow lesions, cartilage thickness and surface area, effusion-synovitis, meniscus morphology and -extrusion, osteophyte size, and Hoffa-synovitis, change over 24 months was associated with progression of disease [7]. However, definitions of change using complex scoring systems are challenging and need to be defined carefully prior to engaging in detailed analyses focused on outcomes and prediction models. As currently only sparse data are available on reliability and definitions of change in semi-quantitatively assessed MRI studies, we believe that a detailed description will be helpful to investigators focusing on samples at risk for progression; these data were not covered in the recent publication [7].

Thus, the aims of our study were to describe the scoring methodology and MRI assessments used to evaluate the cross-sectional features observed in cases and controls, to define change over time for different MRI features and to report the extent of changes over a 24-month period, which may serve as a potential reference for future studies focusing on MRI features and progression over similar observational periods.

## Methods

### Study design

The Osteoarthritis Initiative (OAI) is an ongoing multi-center prospective observational cohort study of knee OA (http://www.oai.ucsf.edu/) that enrolled 4796 participants aged 45–79 years at four clinical centers. Clinical data, MRI scans, radiographs and serum and urine specimens were obtained at baseline, 12, 24, 36, and 48 months (M) follow-up [8]. Eligible participants for the present study were those with at least one knee with a Kellgren-Lawrence grade (KLG) of 1–3 at baseline.

### Criteria for case–control selection

Radiographic progression was defined by a decrease in minimal joint space width of ≥0.7 mm in loss in the medial tibio-femoral compartment from baseline to 24, 36 or 48 M.

Knee pain was assessed using the Western Ontario McMasters (WOMAC) pain subscale. Symptomatic progression was defined as a persistent increase of ≥9 points on a 0–100 normalized score from baseline to 24, 36, 48 or 60 months. This difference has been documented to be clinically relevant [9].

For the nested case–control study, a predetermined number of index knees was selected in the following outcome groups for measurement of imaging biomarkers [6]: 1) case knees had both radiographic and pain progression; control knees did not have this combination, and included 2) knees with radiographic but not pain progression, 3) knees with pain but not radiographic progression, and 4) knees with neither radiographic nor pain progression. The sample size for cases and these three control groups was 194, 103, 103 and 200 knees, respectively. For the purposes of this analysis we compared 194 cases vs. 406 controls.

### MRI acquisition and assessment

MRIs of both knees were acquired using 3 T systems (Siemens Trio) at the 4 OAI clinical sites. A dedicated quadrature transmit/receive knee coil was used and the sequence protocol included a coronal intermediate-weighted 2-dimensional turbo spin echo sequence, a sagittal 3-dimensional dual-echo steady-state sequence, and a sagittal intermediate-weighted fat-suppressed turbo spin-echo sequence [10].

Two musculoskeletal radiologists with 13 (FWR) and 15 (AG) years’ experience of semi-quantitative assessment of knee OA, blinded to clinical data and case–control status, read the baseline and 24 month MRIs according to a validated scoring system [11], and with knowledge of the chronological order of the scans. The following joint structures were assessed: cartilage morphology, osteophytes, subchondral bone marrow lesions (BMLs), meniscal structural damage and meniscal extrusion, Hoffa-synovitis and effusion-synovitis.

In addition, within-grade changes were coded that fulfill the definition of a definite visual change but do not fulfill the definition of a full grade change on the ordinal scales applied [12].

### Reliability

One experienced musculoskeletal radiologist (FWR) re-evaluated 20 randomly selected MRIs in random order after a 4 week interval to assess intra-reader reliability. Inter-observer reliability between the two readers was determined using the same 20 cases.

### Definition of change over time

#### BMLs

**Fig. 1:**
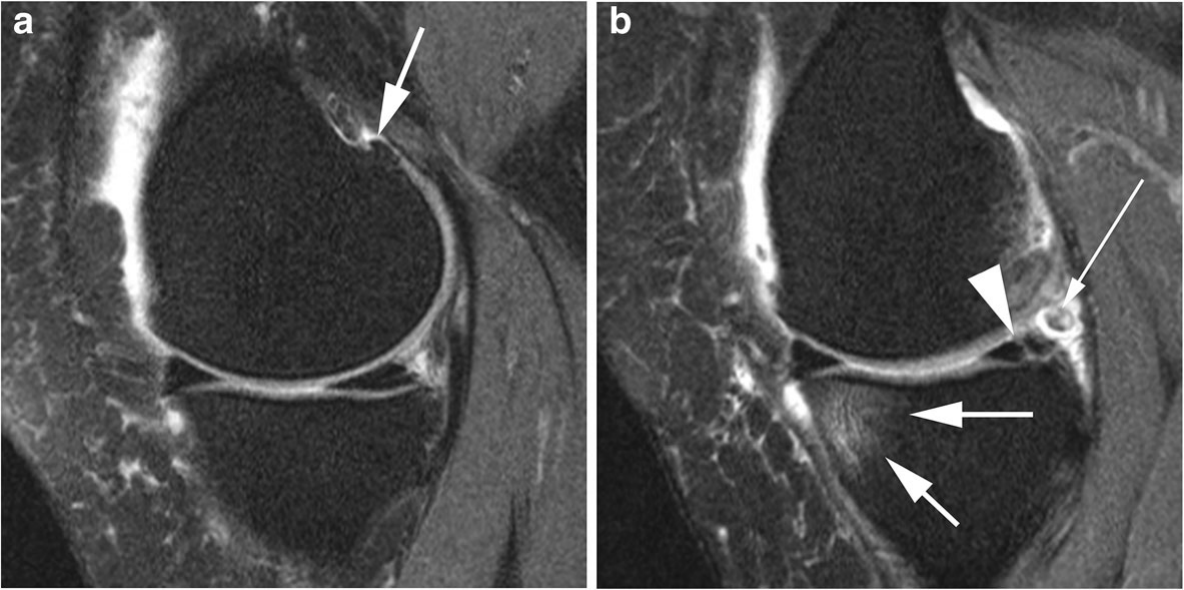
Incident BML and meniscal tear. **a** Baseline sagittal intermediate-weighted fat suppresed image shows normal cartilage coverage of the medial femur and tibia and no meniscal damage. There is a definite osteopyhte at the posterior femur (*arrow*). **b** Follow-up image shows incident BML at the anterior medial tibia (*short, large arrows*) and an incident vertical meniscal tear at the posterior horn of the medial meniscus (*arrowhead*). In addition there is a small loose body posterior to the meniscus (*long, thin arrow*)

Change in overall number of subregions affected by any BML was defined as the difference between the number of subregions affected by any BML at 24 months (size > 0) and the number of subregions affected by any BML at baseline. This was further categorized into improvement, no change, and worsening in one subregion and worsening in two or more subregions. An example of incident BML at follow-up is shown in Fig. 1.

We also determined the number of subregions with worsening, and the number of subregions with improvement. In both instances we took into account within-grade changes in BML size. We further classified these measures into any subregions with worsening vs. no subregions with worsening and any subregions with improvement vs. no subregions with improvement.

To determine maximum change in BML size score, we first evaluated change in size score in each of the 14 articular subregions between baseline and 24 months. Change in size score in each subregion could range from a maximal improvement by three to a maximal worsening by three. The second step was to create an overall change in size score that was defined as the maximum change in size score across the 14 articular subregions. It was categorized into improvement, no change, worsening within grade, worsening by 1 grade, and worsening by two or more grades. Based on distributional quantities the final grouping included: worsening by <2 grades (comprised of improvement, within grade worsening and worsening in at most one grade in size score) vs. worsening by two or more grades.

#### Osteophytes

The change in number of locations affected by any osteophyte was defined as the difference between the number of locations affected by any osteophyte at 24 months (Grade > 0) and the number of locations affected by any osteophyte at baseline. This change was classified as no change, worsening in one location, and worsening by two or more locations and then further classified into no change vs. any worsening. To determine maximum worsening in osteophyte score, we evaluated change in score in each of the 12 locations between baseline and 24 months. Maximum worsening in score was defined as the greatest amount of worsening among the 12 locations. Maximum worsening in score was initially classified as no change, worsening one grade, and worsening by two or more grades. Based on the distribution, the final categorization included no worsening vs. any worsening.

#### Meniscus

**Fig. 2:**
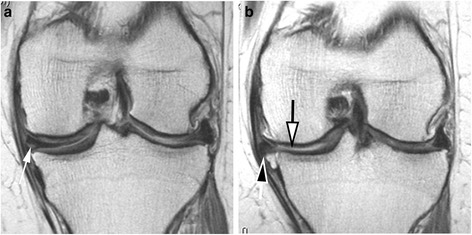
Progression of meniscal damage and incident cartilage loss over 24 months. **a** Baseline coronal intermediate-weighted image shows horizontal-oblique tear of the body of the medial meniscus (*arrow*). There is no apparent cartilage damage at the tibia or femur at the medial compartment. **b** Follow-up image obtained 24 months later shows marked incident meniscal extrusion (*black-filled arrow*) and newly developed cartilage loss at the central portion of the medial tibia (*white-filled arrow*)

We assessed whether there was worsening in meniscal morphology from baseline to 24 months in each of the six meniscal subregions. We defined worsening as an increase in grade in at least one subregion. Figure 2 ﻿shows an example of increase in meniscal extrusion over time. We further categorized worsening in meniscal morphology into number of compartments with worsening (range 0–6) and whether any of the compartments had worsening (yes/no). We assessed changes in meniscal extrusion separately in the medial and lateral compartments. We categorized change in extrusion as improvement, no change, and worsening. We further dichotomized change in extrusion as no worsening vs. any worsening.

#### Cartilage

MOAKS uses a two-digit score for cartilage assessment that incorporates both area size per subregion and percentage of subregion affected by full thickness cartilage loss. In this analysis separate scores for cartilage thickness and surface area were considered. The number of subregions with worsening (i.e., a higher score at 24 months vs. baseline) was defined separately for surface area and thickness. Change over time for surface area was computed in two ways: including within-grade changes and excluding-within grade changes. Within grade scoring for cartilage refers to within grade change in area or thickness. For both thickness and surface area, worsening was grouped into 4-levels: 0, 1, 2, or 3 or more areas with worsening.

#### Hoffa-synovitis and effusion-synovitis

As MRI markers of inflammation so-called effusion- and Hoffa-synovitis are evaluated. Fluid sensitive sequences as applied in the OAI are capable of delineating intraarticular joint fluid but a distinction between true joint effusion and synovial thickening is not possible as both are visualized as hyperintense signal within the joint cavity. For this reason the term effusion-synovitis has been introduced, which is scored based on the distension of the joint capsule. Hoffa-synovitis is a term used for signal changes in Hoffa’s fat pad that are commonly used as a surrogate for synovitis on non-contrast enhanced MRI. Effusion-synovitis is scored from 0 to 3 according to the distention of the joint capsule as 1 = small, 2 = moderate and 3 = large. Hoffa-synovitis is scored based on the amount of hyperintensity signal in Hoffa’s fat pad on sagittal fat suppressed intermediate-weighted sequences as 1 = mild, 2 = moderate and 3 = severe.

Twenty-four month changes in both, Hoffa-synovitis and effusion-synovitis were categorized as improvement, no change, or worsening.

### Analytic approach

Descriptive statistics were used to report frequencies for the different features and parameters for baseline and change over time. Logistic regression was used to identify factors associated with statistically significant differences between cases and controls. For some features raw distributions were grouped into categories as described above. In these instances descriptive statistics are presented for both raw and categorical versions of features, and regression was used only for the categorical version. Weighted kappa statistics were applied to determine inter- and intra-observer reliability. All analyses were conducted in SAS 9.4 (SAS Institute, Cary NC).

## Results

Mean age of the participants was 62 years, 60 % were women and average BMI was 30 kg/m^2^ [5]. Cases and controls were balanced on all covariates, with the exception of baseline KLG with a higher proportion of KL3 knees in the case group (44 %) compared to the controls (33 %). Summarizing the intra- and inter-observer reliability results, all of the measures showed at least substantial agreement ranging between 0.68 for Hoffa-synovitis and 0.97 for medial and lateral meniscal morphology. Table 1 gives a detailed overview of the reliability results.

**Table 1:** Intra- and inter-observer · reliability

		Weighted kappa	Standard error	95 % confidence interval		Weighted kappa	Standard error	95 % confidence interval	
				Lower	Upper			Lower	Upper
		Intra-observer reliability				Inter-observer reliability			
Cartilage area involvement (0–3)	Whole knee	0.87	0.054	0.76	0.97	0.89	0.053	0.78	0.99
	Medial TFJ	0.84	0.092	0.66	1.00	0.87	0.090	0.69	1.00
	Lateral TFJ	0.89	0.092	0.71	1.00	0.96	0.093	0.78	1.00
	PFJ	0.86	0.094	0.68	1.00	0.82	0.094	0.64	1.00
Cartilage % of subregion affected by full thickness damage (0–3)	Whole knee	0.78	0.056	0.67	0.89	0.86	0.055	0.75	0.96
	Medial TFJ	0.73	0.091	0.56	0.91	0.84	0.095	0.65	1.00
	Lateral TFJ	0.73	0.096	0.54	0.92	0.79	0.093	0.61	0.97
	PFJ	0.85	0.101	0.65	1.00	0.92	0.100	0.73	1.00
BML size (0–3)	Whole knee	0.87	0.050	0.77	0.97	0.90	0.050	0.80	1.00
	Medial TFJ	0.84	0.081	0.68	1.00	0.90	0.082	0.74	1.00
	Lateral TFJ	0.90	0.087	0.73	1.00	0.90	0.086	0.73	1.00
	PFJ	0.87	0.093	0.69	1.00	0.89	0.092	0.71	1.00
Meniscus Morphology (0–8)	Whole knee	0.97	0.085	0.81	1.00	0.99	0.085	0.82	1.00
	Medial TFJ	0.97	0.112	0.74	1.00	1.00	0.120	0.76	1.00
	Lateral TFJ	0.97	0.121	0.73	1.00	0.97	0.121	0.73	1.00
Meniscus Extrusion^a^(0–3)	Medial	0.83	0.226	0.38	1.00	0.58	0.208	0.17	0.99
	Lateral	0.88	0.228	0.44	1.00	0.76	0.222	0.32	1.00
Hoffa-Synovitis (0–3)		0.68	0.157	0.38	0.99	0.68	0.157	0.38	0.99
Effusion-Synovitis (0–3)		0.95	0.174	0.61	1.00	0.91	0.171	0.57	1.00

^a^Simple kappa for meniscal extrusion: dichotomized into 0 and 1–3 (>2 mm)

### BMLs

The number of sub-regions affected by any BML ranged from zero to eight and the maximum BML score per knee ranged from zero to three. The change in number of subregions affected by any BML ranged from −3 (three fewer subregions affected at 24 months compared to baseline) to 5 (five more subregions affected at 24 months compared to baseline). Fourteen percent of subjects showed improvement in number of subregions with BMLs (fewer subregions with BMLs at 24 months as compared to baseline) and 52 % showed no change based on this definition. Seventy-three percent of the cases had any subregions with worsening (vs. 66 % in the control group).

### Osteophytes

The number of locations with any osteophytes ranged from zero to 12. The maximum osteophyte score per knee was zero for 3 % of knees, one for 48 %, two for 34 % and three for 15 % of the knees. Overall there was very little change in osteophytes over 24 months. Nine percent of the cohort had at least one location that worsened in osteophyte score over 24 months. Across all locations, the maximum amount of worsening was 2 grades (i.e., zero to two or one to three) and 83 % had no change in any location.

### Meniscus

Thirty percent of the knees had any meniscal tear and 28 % showed meniscal substance loss (i.e. maceration). The number of regions with meniscal morphology worsening ranged from zero to five, with 16 % of subjects having worsening in at least one subregion. Fourteen percent showed an increase in medial meniscal extrusion while only one knee had an increase in lateral extrusion.

### Cartilage

The number of subregions with worsening in cartilage surface area, including within-grade changes, ranged from zero to eight with 59 % of subjects having at least one area with worsening in surface area. The number of subregions with cartilage thickness score > 0 ranged from zero to seven. Across the entire knee, the number of areas with worsening in cartilage thickness ranged from zero to six with 42 % of subjects having at least one area with worsening in thickness.

### Hoffa-synovitis

MOAKS Hoffa-synovitis score ranged from zero to seven and with 24 month change ranging from −2 to 2. While only 10 % of subjects experienced worsening, more cases experienced worsening than controls (17 % vs. 6 %).

### Effusion-synovitis

MOAKS effusion-synovitis score ranged from zero to three with 24 month changes ranging from −2 to 2. Forty-one percent of cases worsened compared to 18 % of controls.

Apart from meniscal damage and effusion-synovitis, baseline frequencies of all measures showed statistically significant differences for cases vs. controls. For change parameters, maximum worsening of BML score, 24 months change in osteophytes and meniscal damage and extrusion, all cartilage measures, and Hoffa- and effusion-synovitis showed significant differences between cases and controls.

Tables 2, 3 and 4 present the baseline frequencies of BMLs, osteophytes and the menisci including the grouping of the different scores into broader summary categories, while Tables 5 and 6 show in detail the frequencies for cartilage, Hoffa- and effusion-synovitis. The change observations for the different features are presented in detail in Tables 7, 8, 9 and 10.

**Table 2:** Baseline frequencies of semi-quantitative MRI biomarkers – BMLs

MRI feature	Biomarker	Overall *n* (%)	Controls	Cases	*p*-value
BML	Number of subregions affected by any BML				
	0	66 (11 %)	55 (13.5 %)	11 (5.7 %)	
	1	98 (16 %)	76 (18.7 %)	22 (11.3 %)	
	2	126 (21 %)	88 (21.7 %)	38 (19.6 %)	
	3	129 (22 %)	88 (21.7 %)	41 (21.1 %)	
	4	84 (14 %)	52 (12.8 %)	32 (16.5 %)	
	5	59 (10 %)	27 (6.7 %)	32 (16.5 %)	
	6	26 (4 %)	14 (3.4 %)	12 (6.2 %)	
	7	8 (1 %)	5 (1.2 %)	3 (1.5 %)	
	8	4 (1 %)	1 (0.2 %)	3 (1.5 %)	
	Number of subregions affected by any BML				<0.001*
	0	66 (11 %)	55 (13.5 %)	11 (5.7 %)	
	1	98 (16 %)	76 (18.7 %)	22 (11.3 %)	
	2	126 (21 %)	88 (21.7 %)	38 (19.6 %)	
	3	129 (22 %)	88 (21.7 %)	41 (21.1 %)	
	4	84 (14 %)	52 (12.8 %)	32 (16.5 %)	
	5+	97 (16 %)	47 (11.6 %)	50 (25.8 %)	
	Max BML score in knee				0.016*
	0	66 (11 %)	55 (13.5 %)	11 (5.7 %)	
	1	223 (37 %)	148 (36.5 %)	75 (38.7 %)	
	2	202 (34 %)	138 (34.0 %)	64 (33.0 %)	
	3	109 (18 %)	65 (16.0 %)	44 (22.7 %)	

*statistically significant at *p* < 0.05; *p* values refer to differences between cases and controls across all grades

**Table 3:** Baseline frequencies of semi-quantitative MRI biomarkers – osteophytes

MRI feature	Biomarker	Overall *n* (%)	Controls	Cases	*p*-value
Osteophytes	Number of locations affected by any osteophyte				
	0	19 (3 %)	15 (3.7 %)	4 (2.1 %)	
	1	34 (6 %)	30 (7.4 %)	4 (2.1 %)	
	2	42 (7 %)	36 (8.9 %)	6 (3.1 %)	
	3	58 (10 %)	43 (10.6 %)	15 (7.7 %)	
	4	54 (9 %)	39 (9.6 %)	15 (7.7 %)	
	5	47 (8 %)	38 (9.4 %)	9 (4.6 %)	
	6	45 (8 %)	28 (6.9 %)	17 (8.8 %)	
	7	37 (6 %)	23 (5.7 %)	14 (7.2 %)	
	8	49 (8 %)	26 (6.4 %)	23 (11.9 %)	
	9	41 (7 %)	26 (6.4 %)	15 (7.7 %)	
	10	47 (8 %)	30 (7.4 %)	17 (8.8 %)	
	11	48 (8 %)	33 (8.1 %)	15 (7.7 %)	
	12	79 (13 %)	39 (9.6 %)	40 (20.6 %)	
	Number of locations affected by any osteophyte category				<0.001*
	0–2	95 (16 %)	81 (20.0 %)	14 (7.2 %)	
	3–5	159 (27 %)	120 (29.6 %)	39 (20.1 %)	
	6+	346 (58 %)	205 (50.5 %)	141 (72.7 %)	
	Max osteophyte score in knee				
	0	19 (3 %)	15 (3.7 %)	4 (2.1 %)	
	1	290 (48 %)	209 (51.5 %)	81 (41.8 %)	
	2	202 (34 %)	132 (32.5 %)	70 (36.1 %)	
	3	89 (15 %)	50 (12.3 %)	39 (20.1 %)	
	Max osteophyte score in knee category				0.011*
	0–1	309 (52 %)	224 (55.2 %)	85 (43.8 %)	
	2	202 (34 %)	132 (32.5 %)	70 (36.1 %)	
	3	89 (15 %)	50 (12.3 %)	39 (20.1 %)	

*statistically significant at *p* < 0.05; *p* values refer to differences between cases and controls across all grades

**Table 4:** Baseline frequencies of semi-quantitative MRI biomarkers – meniscus

MRI feature	Biomarker	Overall *n* (%)	Controls	Cases	*p*-value
Meniscus	Meniscal Morphology: baseline maximum grade in all meniscal subregions				0.345
	0 (normal or signal only, grades 0 and 1)	253 (42 %)	178 (43.8 %)	75 (38.7 %)	
	1 (any tear, grades 2–5)	179 (30 %)	114 (28.1 %)	65 (33.5 %)	
	2 (any maceration, grades 6–8)	168 (28 %)	114 (28.1 %)	54 (27.8 %)	
	Meniscal extrusion: medial				0.005*
	Grade 0: < 2 mm	202 (34 %)	151 (37.4 %)	51 (26.3 %)	
	Grade 1: 2–2.9 mm	176 (29 %)	119 (29.5 %)	57 (29.4 %)	
	Grade 2: 3–4.9 mm	164 (27 %)	106 (26.2 %)	58 (29.9 %)	
	Grade 3: > 5 mm	56 (9 %)	28 (6.9 %)	28 (14.4 %)	
	Meniscal Extrusion: lateral				
	Grade 0: < 2 mm	577 (96 %)	387 (95.3 %)	190 (97.9 %)	
	Grade 1: 2–2.9 mm	7 (1 %)	5 (1.2 %)	2 (1.0 %)	
	Grade 2: 3–4.9 mm	15 (3 %)	13 (3.2 %)	2 (1.0 %)	
	Grade 3: > 5 mm	1 (0 %)	1 (0.2 %)	0 (0.0 %)	

*statistically significant at *p* < 0.05; *p* values refer to differences between cases and controls across all grades

**Table 5:** Baseline frequencies of semi-quantitative MRI biomarkers – cartilage

MRI feature	Biomarker	Overall *n* (%)	Controls	Cases	*p*-value
Cartilage	MOAKS Cartilage Morphology-Thickness-Max score across entire knee				0.012*
	0	150 (25 %)	118 (29.1 %)	32 (16.5 %)	
	1	127 (21 %)	83 (20.4 %)	44 (22.7 %)	
	2	290 (48 %)	184 (45.3 %)	106 (54.6 %)	
	3	33 (6 %)	21 (5.2 %)	12 (6.2 %)	
	MOAKS Cartilage Morphology-Thickness-Number of subregions with score > 0 across entire knee				
	0	150 (25 %)	118 (29.1 %)	32 (16.5 %)	
	1	169 (28 %)	112 (27.6 %)	57 (29.4 %)	
	2	123 (21 %)	82 (20.2 %)	41 (21.1 %)	
	3	78 (13 %)	46 (11.3 %)	32 (16.5 %)	
	4	54 (9 %)	34 (8.4 %)	20 (10.3 %)	
	5	19 (3 %)	11 (2.7 %)	8 (4.1 %)	
	6	4 (1 %)	2 (0.5 %)	2 (1.0 %)	
	7	3 (1 %)	1 (0.2 %)	2 (1.0 %)	
	MOAKS Cartilage Morphology-Thickness-Number of subregions with score > 0 across entire knee category				0.002*
	0	150 (25 %)	118 (29.1 %)	32 (16.5 %)	
	1,2	292 (49 %)	194 (47.8 %)	98 (50.5 %)	
	3+	158 (26 %)	94 (23.2 %)	64 (33.0 %)	
	MOAKS Cartilage Morphology-Surface Area - Max score across entire knee				
	0	12 (2 %)	12 (3.0 %)	0 (0.0 %)	
	1	28 (5 %)	22 (5.4 %)	6 (3.1 %)	
	2	430 (72 %)	297 (73.2 %)	133 (68.6 %)	
	3	130 (22 %)	75 (18.5 %)	55 (28.4 %)	
	MOAKS Cartilage Morphology-Surface Area - Max score across entire knee Category				0.004*
	0–1	40 (7 %)	34 (8.4 %)	6 (3.1 %)	
	2	430 (72 %)	297 (73.2 %)	133 (68.6 %)	
	3	130 (22 %)	75 (18.5 %)	55 (28.4 %)	
	MOAKS Cartilage Morphology-Surface Area-Number of subregions with score > 0 across entire knee				
	0	12 (2 %)	12 (3.0 %)	0 (0.0 %)	
	1	33 (6 %)	28 (6.9 %)	5 (2.6 %)	
	2	50 (8 %)	34 (8.4 %)	16 (8.2 %)	
	3	78 (13 %)	59 (14.5 %)	19 (9.8 %)	
	4	102 (17 %)	81 (20.0 %)	21 (10.8 %)	
	5	111 (19 %)	69 (17.0 %)	42 (21.6 %)	
	6	88 (15 %)	54 (13.3 %)	34 (17.5 %)	
	7	50 (8 %)	30 (7.4 %)	20 (10.3 %)	
	8	36 (6 %)	18 (4.4 %)	18 (9.3 %)	
	9	23 (4 %)	12 (3.0 %)	11 (5.7 %)	
	10	13 (2 %)	7 (1.7 %)	6 (3.1 %)	
	11	2 (0 %)	1 (0.2 %)	1 (0.5 %)	
	12	1 (0 %)	1 (0.2 %)	0 (0.0 %)	
	13	1 (0 %)	0 (0.0 %)	1 (0.5 %)	
	MOAKS Cartilage Morphology-Surface Area - Number of subregions with score > 0 across entire knee category				<0.001*
	0–1	45 (8 %)	40 (9.9 %)	5 (2.6 %)	
	2–4	230 (38 %)	174 (42.9 %)	56 (28.9 %)	
	5–7	249 (42 %)	153 (37.7 %)	96 (49.5 %)	
	8+	76 (13 %)	39 (9.6 %)	37 (19.1 %)	

*statistically significant at *p* < 0.05; *p* values refer to differences between cases and controls across all grades

**Table 6:** Baseline frequencies of semi-quantitative MRI biomarkers –hoffa- and effusion-synovitis

MRI feature	Biomarker	Overall *n* (%)	Controls	Cases	*p*-value
Hoffa-synovitis					0.004*
	0	246 (41 %)	186 (45.8 %)	60 (30.9 %)	
	1	302 (50 %)	190 (46.8 %)	112 (57.7 %)	
	2	47 (8 %)	26 (6.4 %)	21 (10.8 %)	
	3	5 (1 %)	4 (1.0 %)	1 (0.5 %)	
Effusion-synovitis					0.560
	0	233 (39 %)	156 (38.4 %)	77 (39.7 %)	
	1	250 (42 %)	176 (43.3 %)	74 (38.1 %)	
	2	97 (16 %)	62 (15.3 %)	35 (18.0 %)	
	3	20 (3 %)	12 (3.0 %)	8 (4.1 %)	

*statistically significant at *p* < 0.05; *p* values refer to differences between cases and controls across all grades

**Table 7:** Twenty-four month change in semi-quantitative MRI biomarkers – BMLs

MRI feature	Biomarker	Overall *n* (%)	Controls	Cases	*p*-value
BML	Change in Number of subregions affected by any BML (BL to 24 M)				
	-3	4 (1 %)	3 (0.7 %)	1 (0.5 %)	
	-2	6 (1 %)	4 (1.0 %)	2 (1.0 %)	
	-1	71 (12 %)	48 (11.9 %)	23 (11.9 %)	
	0	309 (52 %)	214 (52.8 %)	95 (49.0 %)	
	1	154 (26 %)	105 (25.9 %)	49 (25.3 %)	
	2	43 (7 %)	24 (5.9 %)	19 (9.8 %)	
	3	9 (2 %)	7 (1.7 %)	2 (1.0 %)	
	4	2 (0 %)	0 (0.0 %)	2 (1.0 %)	
	5	1 (0 %)	0 (0.0 %)	1 (0.5 %)	
	Change in Number of subregions affected by any BML (BL to 24 M)				0.318
	Improvement	81 (14 %)	55 (13.6 %)	26 (13.4 %)	
	No Change	309 (52 %)	214 (52.8 %)	95 (49.0 %)	
	Worsen in 1 subregion	154 (26 %)	105 (25.9 %)	49 (25.3 %)	
	Worsen in 2+ subregions	55 (9 %)	31 (7.7 %)	24 (12.4 %)	
	Maximum worsening in BML score of all subregions in knee (BL to 24 M)				0.003*
	0	191 (32 %)	138 (34.1 %)	53 (27.3 %)	
	1	36 (6 %)	24 (5.9 %)	12 (6.2 %)	
	2	273 (46 %)	192 (47.4 %)	81 (41.8 %)	
	3	99 (17 %)	51 (12.6 %)	48 (24.7 %)	
	Number of subregions with any improvement in BML (including within grade changes – BL to 24 months)				
	0	308 (51 %)	219 (53.9 %)	89 (45.9 %)	
	1	192 (32 %)	132 (32.5 %)	60 (30.9 %)	
	2	76 (13 %)	42 (10.3 %)	34 (17.5 %)	
	3	18 (3 %)	11 (2.7 %)	7 (3.6 %)	
	4	4 (1 %)	2 (0.5 %)	2 (1.0 %)	
	5	1 (0 %)	0 (0.0 %)	1 (0.5 %)	
	6	1 (0 %)	0 (0.0 %)	1 (0.5 %)	
	Any subregions with improvement (including within grade changes) in BML				0.065
	No	308 (51 %)	219 (53.9 %)	89 (45.9 %)	
	Yes	292 (49 %)	187 (46.1 %)	105 (54.1 %)	
	Number of subregions with any worsening (including within grade changes) in BML				
	0	191 (32 %)	138 (34.0 %)	53 (27.3 %)	
	1	194 (32 %)	143 (35.2 %)	51 (26.3 %)	
	2	128 (21 %)	79 (19.5 %)	49 (25.3 %)	
	3	53 (9 %)	31 (7.6 %)	22 (11.3 %)	
	4	23 (4 %)	9 (2.2 %)	14 (7.2 %)	
	5	10 (2 %)	6 (1.5 %)	4 (2.1 %)	
	6	1 (0 %)	0 (0.0 %)	1 (0.5 %)	
	Any subregions with worsening (including within grade changes) in BML				0.102
	No	191 (32 %)	138 (34.0 %)	53 (27.3 %)	
	Yes	409 (68 %)	268 (66.0 %)	141 (72.7 %)	

*statistically significant at *p* < 0.05; *p* values refer to differences between cases and controls across all grades

**Table 8:** Twenty-four month change in semi-quantitative MRI biomarkers – osteophytes

MRI feature	Biomarker	Overall *n* (%)	Controls	Cases	*p*-value
Osteophytes	24 M Increase in number of locations affected by any osteophyte				0.386
	No	544 (91 %)	371 (91.4 %)	173 (89.2 %)	
	Yes	56 (9 %)	35 (8.6 %)	21 (10.8 %)	
	24 M Change in number of locations affected by any Osteophyte				0.585
	No Change	544 (91 %)	371 (91.4 %)	173 (89.2 %)	
	Worsen in 1 subregion	32 (5 %)	21 (5.2 %)	11 (5.7 %)	
	Worsen in 2+ subregions	24 (4 %)	14 (3.4 %)	10 (5.2 %)	
	24 M Max change in osteophyte score across all locations in knee				
	0	498 (83 %)	347 (85.5 %)	151 (77.8 %)	
	1	97 (16 %)	55 (13.5 %)	42 (21.6 %)	
	2	5 (1 %)	4 (1.0 %)	1 (0.5 %)	
	24 M Max change in osteophyte score > =1 across all locations in knee				0.021*
	No	498 (83 %)	347 (85.5 %)	151 (77.8 %)	
	Yes	102 (17 %)	59 (14.5 %)	43 (22.2 %)	

*statistically significant at *p* < 0.05; *p* values refer to differences between cases and controls across all grades

**Table 9:** Twenty-four month change in semi-quantitative MRI biomarkers – meniscus

MRI feature	Biomarker	Overall *n* (%)	Controls	Cases	*p*-value
	Yes	102 (17 %)	59 (14.5 %)	43 (22.2 %)	
Meniscus	Meniscal Morphology: 24 Month Number of regions with worsening				
	0	505 (84 %)	365 (90.1 %)	140 (72.2 %)	
	1	67 (11 %)	31 (7.7 %)	36 (18.6 %)	
	2	25 (4 %)	7 (1.7 %)	18 (9.3 %)	
	3	1 (0 %)	1 (0.2 %)	0 (0.0 %)	
	5	1 (0 %)	1 (0.2 %)	0 (0.0 %)	
	Meniscal Morphology: 24 Month Any regions with worsening				<0.001
	No	505 (84 %)	365 (90.1 %)	140 (72.2 %)	
	Yes	94 (16 %)	40 (9.9 %)	54 (27.8 %)	
	Meniscal Extrusion Medial - 24 Month worsening				<0.001
	No	512 (86 %)	369 (91.3 %)	143 (74.1 %)	
	Yes	85 (14 %)	35 (8.7 %)	50 (25.9 %)	
	Meniscal Extrusion Lateral - 24 Month worsening				
	No	599 (100 %)	405 (99.8 %)	194 (100.0 %)	
	Yes	1 (0 %)	1 (0.2 %)	0 (0.0 %)	

*statistically significant at *p* < 0.05; *p* values refer to differences between cases and controls across all grades

**Table 10:** Twenty-four month change in semi-quantitative MRI biomarkers – cartilage

MRI feature	Biomarker	Overall *n* (%)	Controls	Cases	*p*-value
Cartilage	MOAKS Cartilage Morphology - entire knee number of areas with BL to 24 M worsening in thickness				
	0	348 (58 %)	266 (65.5 %)	82 (42.3 %)	
	1	132 (22 %)	83 (20.4 %)	49 (25.3 %)	
	2	77 (13 %)	39 (9.6 %)	38 (19.6 %)	
	3	34 (6 %)	13 (3.2 %)	21 (10.8 %)	
	4	8 (1 %)	5 (1.2 %)	3 (1.5 %)	
	6	1 (0 %)	0 (0.0 %)	1 (0.5 %)	
	MOAKS Cartilage Morphology - entire knee number of areas with BL to 24 M worsening in thickness				<0.001
	No Change	348 (58 %)	266 (65.5 %)	82 (42.3 %)	
	Worsen in 1 subregion	132 (22 %)	83 (20.4 %)	49 (25.3 %)	
	Worsen in 2 subregions	77 (13 %)	39 (9.6 %)	38 (19.6 %)	
	Worsen in 3+ subregions	43 (7 %)	18 (4.4 %)	25 (12.9 %)	
	MOAKS Cartilage Morphology - entire knee number of areas with BL to 24 M worsening in surface area (include within-grade change)				
	0	246 (41 %)	193 (47.5 %)	53 (27.3 %)	
	1	176 (29 %)	122 (30.0 %)	54 (27.8 %)	
	2	91 (15 %)	52 (12.8 %)	39 (20.1 %)	
	3	60 (10 %)	31 (7.6 %)	29 (14.9 %)	
	4	20 (3 %)	6 (1.5 %)	14 (7.2 %)	
	5	5 (1 %)	2 (0.5 %)	3 (1.5 %)	
	6	1 (0 %)	0 (0.0 %)	1 (0.5 %)	
	8	1 (0 %)	0 (0.0 %)	1 (0.5 %)	
	MOAKS Cartilage Morphology - entire knee number of areas with BL to 24 M worsening in surface area (include within-grade change)				<0.001
	No Change	246 (41 %)	193 (47.5 %)	53 (27.3 %)	
	Worsen in 1 subregion	176 (29 %)	122 (30.0 %)	54 (27.8 %)	
	Worsen in 2 subregions	91 (15 %)	52 (12.8 %)	39 (20.1 %)	
	Worsen in 3+ subregions	87 (15 %)	39 (9.6 %)	48 (24.7 %)	
	MOAKS Cartilage Morphology - entire knee number of areas with BL to 24 M worsening in surface area (exclude within-grade change)				
	0	382 (64 %)	277 (68.2 %)	105 (54.1 %)	
	1	128 (21 %)	87 (21.4 %)	41 (21.1 %)	
	2	56 (9 %)	31 (7.6 %)	25 (12.9 %)	
	3	28 (5 %)	9 (2.2 %)	19 (9.8 %)	
	4	3 (1 %)	2 (0.5 %)	1 (0.5 %)	
	5	2 (0 %)	0 (0.0 %)	2 (1.0 %)	
	6	1 (0 %)	0 (0.0 %)	1 (0.5 %)	
	MOAKS Cartilage Morphology - entire knee number of areas with BL to 24 M worsening in surface area (exclude within-grade change)				<0.001
	No Change	382 (64 %)	277 (68.2 %)	105 (54.1 %)	
	Worsen in 1 subregion	128 (21 %)	87 (21.4 %)	41 (21.1 %)	
	Worsen in 2 subregions	56 (9 %)	31 (7.6 %)	25 (12.9 %)	
	Worsen in 3+ subregions	34 (6 %)	11 (2.7 %)	23 (11.9 %)	

*statistically significant at *p* < 0.05; *p* values refer to differences between cases and controls across all grades

## Discussion

In this cohort of subjects at risk for OA progression, the values for several tissue-specific MRI features associated with progression of disease vary widely and show great change or fluctuation. The subgroup defined as cases based on composite progression of structural and clinical features exhibited changes to a greater extent than the controls on several features. Specifically, we observed greater change in the case group on maximum change in BMLs, worsening of BMLs in two or more subregions, worsening of cartilage surface area and thickness in three or more subregions and worsening of meniscal damage. Inflammatory markers of disease, i.e. Hoffa- and effusion-synovitis, also worsened more frequently in the case group compared to the controls emphasizing the potential role of inflammation in disease progression [13–15]. Overall little change was observed for osteophytes reflecting the generally slow course of the disease.

Focusing on the identical dataset, we could show using a multivariable approach that 24-month change in cartilage thickness, cartilage surface area, synovitis-effusion, Hoffa-synovitis, and meniscal morphology were associated with disease progression independently, suggesting that they may serve as efficacy biomarkers in clinical trials of disease modifying interventions for knee OA [7]. Definition of change using semi-quantitative approaches is challenging as there are multiple possible definitions including subregional or maximum-grade approaches. To gain additional understanding of frequencies and categories encountered in this cohort selected on the basis of progression or serving as controls we performed the current analysis that may help researchers in the future to power planned observational studies or clinical trials.

Few studies are available that have focused on longitudinal change of MRI parameters using semi-quantitative assessment. Most available studies are centered around baseline predictors of subsequent cartilage loss as the outcome [16]; only few studies focus on cartilage as a predictor of worsening BMLs as the outcome [17]. When assessing change using semi-quantitative scoring in OA, scores are commonly presented as mean values or summed over a defined anatomical region (commonly compartment or knee) [18, 19]. For several reasons, such approaches have drawbacks that need to be considered. One of the main shortcomings is that sums are challenging to compare. As an example, a sum of six acquired over six distinct subregions may mean one lesion with a grade 6 (considered severe) while five other subregions exhibit no lesion (grade 0); alternatively, it may reflect grade 1 lesions in all six subregions. More work is needed on the prognostic implications of having widespread low grade involvement vs. a focal severe lesion. It appears likely that both play a role with regard to disease progression [3]. Other approaches to define progression have been published recently [20].

Part of the study design was sequential reading of MRIs not blinded to time point but blinded to case or control status as it has been shown that this approach increases sensitivity to change [21]. Reading unblinded to time point also allowed for the application of within-grade changes, further increasing sensitivity to detect minor changes [12]. In assessing MRI data semi-quantitatively, we are advocating the scoring of the number of subregions or locations involved by pathology, with further stratification using cut-offs related to severity of a certain feature. In addition, an approach assessing a maximum change over a pre-defined unit, such as a knee compartment or the entire joint, adds to the understanding of the degree of change observed, which may be lost using a summative approach. Our definition of controls included both non-progressors and non-composite progressors including those that either progressed clinically (but not radiographically) or radiographically (but not clinically). A further subanalysis is needed to look at differences in changes for these subgroups separately.

## Conclusions

In summary, a wide range of MRI-detected structural pathologies was present in the FNIH cohort. More severe changes, especially for BMLs, cartilage and meniscal damage, were detected primarily among the case group suggesting that early changes in multiple structural domains are associated with radiographic worsening and symptomatic progression. Particularly the role of structural predictors of progression that are potentially amenable to therapeutic approaches such as inflammatory markers of disease (depicted as Hoffa- and effusion synovitis on MRI) or subchondral bone changes (visualized as BMLs on MRI) should be the focus of further evaluation. In addition, the complexity of the different semi-quantitative scoring systems needs consideration when engaging in analyses focusing on change over time. Simply summing scores does not seem to be sufficient and further validation of analyses taking into account potentially improving features or within-grade scoring is urgently needed to take full advantage of the richness of semi-quantitative data that is considered complementary to more quantitative approaches based on segmentation of 3D datasets.

## Abbreviations

BMI: Body mass index
BMLs: Bone marrow lesions
FNIH: Foundation for the National Institutes of Health
KLG: Kellgren-Lawrence grade
MOAKS: MRI Osteoarthritis Knee Score
MRI: Magnetic resonance imaging
OA: Osteoarthritis
OAI: Osteoarthritis Initiative
WOMAC: Western Ontario and McMaster Universities Osteoarthritis Index
